# Production and characterization of a novel antifungal chitinase identified by functional screening of a suppressive-soil metagenome

**DOI:** 10.1186/s12934-017-0634-8

**Published:** 2017-01-31

**Authors:** Francesca Berini, Ilaria Presti, Fabrizio Beltrametti, Marco Pedroli, Kjell M. Vårum, Loredano Pollegioni, Sara Sjöling, Flavia Marinelli

**Affiliations:** 1 Department of Biotechnology and Life Sciences, University of Insubria, Varese, Italy; 2 “The Protein Factory Research Center”, Politecnico di Milano and University of Insubria, Varese, Italy; 3 Actygea, Gerenzano, Varese, Italy; 4 NOBIPOL, Department of Biotechnology, Norwegian University of Science and Technology, Trondheim, Norway; 5 School of Natural Sciences, Technology and Environmental Studies, Södertörn University, Huddinge, Sweden; 6 Chemo Biosynthesis, Corana, Pavia, Italy

**Keywords:** Antifungal chitinase, Functional metagenomics, Heterologous expression, Inclusion bodies, Protein purification, Mode of action

## Abstract

**Background:**

Through functional screening of a fosmid library, generated from a phytopathogen-suppressive soil metagenome, the novel antifungal chitinase—named Chi18H8 and belonging to family 18 glycosyl hydrolases—was previously discovered. The initial extremely low yield of Chi18H8 recombinant production and purification from *Escherichia coli* cells (21 μg/g cell) limited its characterization, thus preventing further investigation on its biotechnological potential.

**Results:**

We report on how we succeeded in producing hundreds of milligrams of pure and biologically active Chi18H8 by developing and scaling up to a high-yielding, 30 L bioreactor process, based on a novel method of mild solubilization of *E. coli* inclusion bodies in lactic acid aqueous solution, coupled with a single step purification by hydrophobic interaction chromatography. Chi18H8 was characterized as a Ca^2+^-dependent mesophilic chitobiosidase, active on chitin substrates at acidic pHs and possessing interesting features, such as solvent tolerance, long-term stability in acidic environment and antifungal activity against the phytopathogens *Fusarium graminearum* and *Rhizoctonia solani*. Additionally, Chi18H8 was found to operate according to a non-processive endomode of action on a water-soluble chitin-like substrate.

**Conclusions:**

Expression screening of a metagenomic library may allow access to the functional diversity of uncultivable microbiota and to the discovery of novel enzymes useful for biotechnological applications. A persisting bottleneck, however, is the lack of methods for large scale production of metagenome-sourced enzymes from genes of unknown origin in the commonly used microbial hosts. To our knowledge, this is the first report on a novel metagenome-sourced enzyme produced in hundreds-of-milligram amount by recovering the protein in the biologically active form from recombinant *E. coli* inclusion bodies.

**Electronic supplementary material:**

The online version of this article (doi:10.1186/s12934-017-0634-8) contains supplementary material, which is available to authorized users.

## Background

The existing repertoire of microbial-derived medically, agriculturally and industrially useful enzymes, and natural products, mostly originated from readily cultivable microorganisms. However, the vast majority of microorganisms in the environment is not cultivable under standard laboratory conditions, making this pool relatively untapped. For example, one gram of land soil may contain 10^10^ microbial cells [1] with an estimated species diversity from 10^4^ [2] to nearly 10^7^ species [3]. Today, metagenomics, that is the sequence- and/or function-based analysis of the collective genome assemblages of organisms, may provide a cultivation-independent access to such diversity and potential [4, 5].

We recently applied different genetic and/or activity-based screenings to metagenomes from suppressive and/or chitin-amended agricultural soils to access novel bacterial chitinolytic enzymes (CEs) [6–8]. After lignocellulose, chitin is the most abundant biopolymer in nature, widely distributed within exoskeletons of insects, fungal cell walls, marine diatoms, shells of crustaceans, eggs of nematodes and zooplankton [9, 10]. For example, there is a chitin and glycoprotein layer—the peritrophic matrix—that lines the midgut of most invertebrates [11]. Chitin is rather resistant to degradation and without the action of microbial CEs, it would be trapped in biomass as insoluble. Actually, bacterial CEs are crucial in the global biogeochemical recycling of carbon and nitrogen through the hydrolysis of chitin. CEs, including endochitinases (EC 3.2.1.14), chitobiosidases (EC 3.2.1.29), *N*-acetyl glucosaminidases (EC 3.2.1.30), chitin deacetylases (EC 3.5.1.14), and chitosanases (EC 3.2.1.132), process the unbranched biopolymer, which is composed of repeated units of *N*-acetylglucosamine (GlcNAc), in different synergic modes [10, 12]. Notably, CEs are attracting an increasing interest because of their potential in biotechnological applications [12–14]: e.g. as biocontrol agents that antagonize chitin-containing phytopathogenic fungi, insects and nematodes in integrated pest management strategies [7, 11, 13], or as industrial biocatalysts for the production of chitin derivatives that possess interesting nutraceutical and pharmaceutical properties [14]. For example, chitosan and chitooligosaccharides (COs) have been used for drug and gene delivery [15, 16]. COs have also been suggested to have a role as signal molecules in antagonism phenomena [7, 11, 13]. The mode of action of CEs is thus important in relation to the length, degree of acetylation and sequence of the COs they generate [17].

Several studies, both on aquatic and soil habitats, have specifically reported on the abundance of CE-encoding genes [6, 18–23]. To date, however, microbial CEs have been identified and isolated mostly by conventional molecular and/or functional screenings of microbial isolates from different environmental samples [24, 25]. Only very recently, the use of metagenomic tools has allowed the identification and isolation of novel bacterial CEs from naturally suppressive or chitin amended agricultural soil through expression screening [7], sequence screening [8] and gene synthesis [26]. In non-naturally suppressive soils, chitin amendment helps to enhance the suppressiveness against soil-borne pathogens by stimulating active chitinolytic microbial communities [22, 27–29].

Through expression screening of a fosmid library generated from a metagenome of a naturally suppressive field soil, we recently isolated the novel bacterial chitinase Chi18H8, which showed less than 45% amino acid sequence identity with known chitinases [7]. Sequence analysis revealed the presence of the consensus sequence of the 18 glycosyl hydrolases (GH) family (including the conserved active site’s motif, DXDXE of the catalytic domain) [30]. Unlike most of the other characterized chitinases of the 18GH family, Chi18H8 seemed to exert antagonistic activity against fungi, coherently with the suppressive nature of the soil from which it was isolated, envisaging its potential use as a biocontrol agent for crop protection [7]. In fact, when the recombinant *Escherichia coli* pGEX-6P-3::*chi18H8* (expressing the novel chitinase) was co-cultivated with phytopathogenic fungi, it inhibited fungal growth. However, the extremely low production and purification yield of Chi18H8 (21 μg/g cell) at that time prevented any further investigation on its properties including its potential biotechnological applications.

Here we report how we succeeded in producing hundreds of milligrams of pure and biologically active Chi18H8 by developing and scaling up a high-yielding process in a 30 L bioreactor, based on the recovery of the recombinant protein from *E. coli* inclusion bodies (IBs). To our knowledge, this is the first report on a bacterial chitinase isolated through functional metagenomics brought to pre-industrial scale production.

## Methods

### *chi18H8* cDNA sub-cloning

The nucleotide sequence of the chitinase Chi18H8 gene was previously deposited in the GenBank database under the accession number KC763366 [7]. The cDNA encoding for the chitinase was sub-cloned into the pET24b(+) expression plasmid (kanamycin resistance; Novagen Inc., Madison, USA) by using the primers chiEcoF (5′-ATAAAGAATTCCATGCGCCAGCTCACGCTTCTC-3′) and chiXhoR (5′-ATAAACTCGAGCTAATTGCCCCTATGCAGACTGG-3′), containing the underlined restriction sites for *Eco*RI and *Xho*I, respectively. The plasmid pGEX-6P-3::*chi18H8*, previously prepared [7], was used as DNA template. *E. coli* DH5α (Invitrogen-Life Technologies, Carlsbad, USA) was used as host for the sub-cloning procedures. The construct, following its control by DNA sequencing (BMR Genomics, Padua, Italy), was transformed into *E. coli* BL21 Star™(DE3) (Invitrogen-Life Technologies). Recombinant *E. coli* strains were maintained on Luria-Bertani broth (LB, Miller’s modification; Sigma-Aldrich, St. Louis, USA) agar plates supplemented with 50 µg/mL kanamycin.

### Chi18H8 expression

All medium components and reagents were from Sigma-Aldrich, unless otherwise stated. Protein expression was carried out in the following media, supplemented with 50 µg/mL kanamycin: LB; terrific broth (TB); super broth (SB: 32 g/L tryptone, 20 g/L yeast extract, 5 g/L NaCl); autoinduction media A and B. Autoinduction medium A composition was based on [31]. Autoinduction medium B included: 10 g/L tryptone, 5 g/L yeast extract, 3.3 g/L (NH_4_)_2_SO_4_, 6.8 g/L KH_2_PO_4_, 7.1 g/L Na_2_HPO_4_, 0.5 g/L glucose, 2 g/L α-lactose, 0.15 g/L MgSO_4_, 2 mg/L CaCl_2_, 2 mg/L MnSO_4_ × H_2_O, 2 mg/L ZnSO_4_, 2 mg/L CoCl_2_, 2 mg/L CuCl_2_ × 2H_2_O, 2 mg/L NiCl_2_, 2 mg/L NH_4_MoO_4_, 2 mg/L FeCl_3_. Trace element (MgSO_4_, CaCl_2_, MnSO_4_ × H_2_O, ZnSO_4_, CoCl_2_, CuCl_2_ × 2H_2_O, NiCl_2_, NH_4_MoO_4_, FeCl_3_) stock solutions were sterilized by filtration (0.2 µm) and stored at 4 °C.

Starter cultures were prepared from a single recombinant *E. coli* colony inoculated in 10 mL LB medium supplemented with 50 µg/mL kanamycin, grown overnight at 37 °C and 200 revolutions per minute (rpm). Baffled 300 mL Erlenmeyer flasks containing 50 mL of the different media were inoculated with the starter culture (initial OD_600nm_ = 0.1) and further incubated as above. For LB, TB and SB media, protein expression was induced by adding 0.4 mM isopropyl β-d-thiogalactopyranoside (IPTG) to cells at early- or late-exponential growth phase. After induction, cells were cultured at various temperatures (37, 25 or 20 °C, respectively) at 200 rpm and harvested by centrifugation (1900×*g* for 30 min at 4 °C) at different time intervals. Following centrifugation, total proteins in the supernatants (i.e. the cell-free fermentation broths) were concentrated by 10% (v/v) trichloroacetic acid precipitation and analyzed by sodium dodecyl sulfate-polyacrylamide gel electrophoresis (SDS-PAGE, see below). In parallel, cell pellets were sonicated on ice (3–5 cycles of 30 s each, with a 30-s interval, using a Branson Sonifier 250, Danbury, USA) in phosphate buffer saline (PBS) pH 7.3 (140 mM NaCl, 2.7 mM KCl, 10.1 mM Na_2_HPO_4_, 1.8 mM KH_2_PO_4_) containing 10 µg/mL deoxyribonuclease (DNase), 0.19 mg/mL phenylmethylsulfonylfluoride (PMSF) and 0.7 mg/mL pepstatin. Soluble (cytoplasmic) and insoluble (containing membranes and IBs) cell fractions were then separated by centrifugation at 20,000×*g* for 1 h at 4 °C. Insoluble fractions were re-suspended in a volume of PBS equal to the corresponding cytoplasmic soluble fraction (2 mL/g cell) for successive SDS-PAGE analyses. In every fraction, protein concentration was determined by the Biuret assay [32].

### Chi18H8 production

Flask cultures of recombinant *E. coli* cells, grown overnight in LB medium supplemented with 50 µg/mL kanamycin, were used to inoculate (initial OD_600nm_ = 0.1) 2 L Erlenmeyer flasks (containing 750 mL medium), or 3 L P-100 Applikon glass reactors (Applikon Biotechnology, Delft, The Netherlands) equipped with a AD1030 biocontroller and AD1032 motor (containing 2 L medium), or 30 L Bioengineering (Bioengineering AG, Wald, Switzerland) stirred fermenter (containing 27 L medium). The medium used throughout was LB medium supplemented with 50 µg/mL kanamycin. 2 L Erlenmeyer flasks were incubated at 37 °C and 200 rpm. Growth in fermentors was conducted at 37 °C and stirring at 500 rpm for 3 L bioreactor or at 300 rpm for the 30 L bioreactor, respectively, at constant 1.0 vvm aeration rate and pressure control set at 0.5 bar. Dissolved oxygen (measured as % of the initial pO_2_ value) and pH were monitored using an Ingold polarographic oxygen electrode and a pH meter, respectively. Foam production was monitored through an antifoam sensor and controlled by adding Antifoam SE-15. When cell culture reached an OD_600nm_ = 0.6, protein production was induced by adding 0.4 mM IPTG: cultivation was then prolonged at 20 °C for further 24 h.

### Recovery of IBs and solubilization of Chi18H8

*Escherichia coli* cells were harvested by centrifugation at 3220×*g* for 20 min, washed with sodium chloride-Tris-EDTA (STE buffer: 10 mM Tris–HCl pH 8.0, 1 mM EDTA [ethylenediaminetetraacetic acid], 100 mM NaCl). The different protocols for IB recovery and solubilization are described in Additional file 1: Table S1. In the case of the acid solubilization method, after centrifugation, the cell pellet was re-suspended in 50 mM Tris–HCl pH 8.0, 25% (w/v) sucrose, 1 mM EDTA, and incubated for 30 min at room temperature under vigorous shaking. After sonication on ice (6 cycles of 30 s each, with a 30-s interval), 0.2 M NaCl, 1% (w/v) sodium deoxycholate (DOC) and 1% (v/v) Nonidet P-40 were added. The sample was further incubated as above and centrifuged (20,000×*g* at 4 °C for 30 min). The pellet was washed with 1% (v/v) Triton X-100 and 1 mM EDTA, followed by centrifugation at 12,000×*g* at 4 °C for 10 min; the procedure was repeated twice. IBs were then washed twice with 10 mL/g cell deionized water, with centrifugation at 12,000×*g* at 4 °C for 10 min in-between, and stored overnight at −20 °C. To solubilize Chi18H8 from IBs, the frozen pellet was re-suspended in 10 mL/g cell of 10 or 100 mM HCl or lactic acid and incubated at 37 °C and 200 rpm for 5 h. Insoluble material was removed by centrifugation at 1900×*g* at 4 °C for 5 min. The solubilized protein was then dialyzed overnight against 100 mM sodium acetate buffer pH 5.0 or 100 mM 4-(2-hydroxyethyl)-1-piperazineethanesulfonic acid (HEPES) pH 5.6.

### Chi18H8 purification

Enzyme samples following dialysis were used for testing different chromatographic methods, described in details in Additional file 1: Table S2. Chi18H8 purification/enrichment was achieved by loading the protein sample after dialysis against 100 mM HEPES pH 5.6 onto the weak anionic exchanger Diaion WA11 resin (Resindion s.r.l., Milan, Italy), previously activated with methanol/water (1:1) and then equilibrated with 100 mM HEPES pH 5.6. Pilot experiments were performed with 1 mL resin (wet volume) or, for large scale purification, with 40 mL of WA11 resin (in a 4.5 cm diameter Amicon column loaded at a flow rate of 20 mL/min). The chromatography, run under hydrophobic interaction (HIC) conditions, allowed to fully recover the Chi18H8 protein in the flow-through, whereas other proteins retained by the WA11 resin were then recovered by an isocratic elution using 50% (v/v) ethanol in 100 mM HEPES pH 5.6. The Chi18H8 concentration was estimated using the theoretical extinction coefficient at 280 nm (77,015 M^-1^cm^-1^), based on the amino acid sequence of the protein.

### SDS-PAGE electrophoresis and zymogram analysis

Chi18H8 size, production and solubilization were analyzed by SDS-PAGE with 12% (w/v) polyacrylamide gels [33] and estimated by densitometric analysis (Quantity One, Bio-Rad Laboratories, Hercules, USA) using known amount of His_6_-glycine oxidase (His_6_-GO) from *Bacillus subtilis* [34] as a standard. Chitinolytic activity was detected through zymogram analysis (semi native-PAGE) using 10% (w/v) polyacrylamide gels containing 0.7 mg/mL of carboxymethyl-chitin-remazol brilliant violet (CM-chitin-RBV) (Loewe Biochemica, Suerlach, Germany), as described in [7]. Briefly, the protein samples were diluted in a sample buffer lacking any reducing agent and incubated for 10 min at room temperature. Electrophoresis was conducted at 4 °C in Tris-glycine-SDS running buffer according to standard running conditions. The gel was rinsed twice in 2.5% (v/v) Triton X-100 for 30 min at room temperature to remove SDS and incubated in 100 mM sodium acetate pH 5.0 at 37 °C until the appearance of clear zones indicating chitinolytic activity. The chitinase from *Trichoderma viride* (Sigma-Aldrich) was used as positive control.

### Chi18H8 activity assay

Chi18H8 activity was assayed with the fluorimetric chitooligosaccharide analogues 4-methylumbelliferyl *N*-acetyl-β-d-glucosaminide (4-MU-GlcNAc), 4-methylumbelliferyl *N*,*N’*-diacetyl-β-d-chitobioside (4-MU-(GlcNAc)_2_) and 4-methylumbelliferyl *N,N’,N’’*-triacetyl-β-d-chitotrioside (4-MU-(GlcNAc)_3_) as described previously [7]. Unless otherwise stated, Chi18H8 activity was assayed at 37 °C in 100 mM sodium acetate pH 5.0 [7]. Chitinolytic activity was also determined on colloidal chitin, prepared from chitin flakes from shrimp shells (Sigma-Aldrich), by the colorimetric method previously described [8]. One unit (U) of Chi18H8 activity was defined as the amount of enzyme that released 1 μmol of 4-MU or GlcNAc per min at 37 °C.

### Effect of pH and temperature on Chi18H8 activity

pH influence on Chi18H8 activity on the substrate 4-MU-(GlcNAc)_2_ was determined by the fluorimetric assay in the following buffers (each 100 mM): glycine–HCl (pH 3.0), sodium acetate (pH 4.0 and 5.0), sodium phosphate (pH 6.0 and 7.0), Tris-HCl (pH 8.0) and sodium pyrophosphate (NaPPi, pH 9.0). Optimal temperature for the enzyme activity was determined by the same fluorescent assay, performing the reaction at various temperatures (from 5 to 70 °C). Stability of the enzyme was tested by incubating the recombinant protein at 30 °C at different pHs (in 100 mM sodium acetate pH 5.0 or 100 mM sodium phosphate pH 6.0 and 7.0): after different incubation times (from 0 to 144 h), the residual chitinolytic activity was assayed on 4-MU-(GlcNAc)_2_.

### Effect of metal ions, organic solvents, detergents and other compounds on Chi18H8 activity

The effect of metal ions [Ca^2+^ (CaCl_2_ × 2H_2_O), Cu^2+^ (CuCl_2_ × 2H_2_O), Fe^3+^ (FeCl_3_ × 6H_2_O), K^+^ (KCl), Mg^2+^ (MgCl_2_ × 6H_2_O), Mn^2+^ (MnCl_2_ × 4H_2_O), Ni^2+^ (NiCl_2_ × 6H_2_O), NH_4_ (NH_4_Cl), Zn^2+^ (ZnCl_2_), Co^2+^ (CoCl_2_ × 6H_2_O)], reducing agents [dithiothreitol (DTT), 2-mercaptoethanol], the chelating agent EDTA, detergents [SDS, Triton X-100, Tween-20, DOC, *N*-lauroylsarcosine (NLS), Nonidet-40], organic solvents [ethanol, methanol, propanol, dimethyl sulfoxide (DMSO)], sugars (GlcNAc, chitobiose) and salt (NaCl) on Chi18H8 activity was investigated by adding each compound to the fluorimetric assay mixture. Final concentrations were: 20 mM for metal ions, EDTA and 2-mercaptoethanol, 10 mM for sugars and DTT, 10% (v/v) for organic solvents, and 1% (w/v) for detergents. In each case the residual activity was compared to the activity without additional compounds, set as 100%.

### Circular dichroism

Far-UV CD spectra were recorded at 15 or 30 °C using a Jasco J-715 spectropolarimeter (Jasco, Cremello, Italy) equipped with temperature control, in the 190–250 nm wavelength range. Measurements were carried out in quartz cuvettes of path length 1 mm, employing a protein solution at 0.2 mg/mL in 10 mM HEPES pH 5.4, and corrected for buffer contribution. Secondary structure composition was calculated from deconvolution of the CD spectra with the program k2d3 (http://k2d3.ogic.ca//index.html) [35]. Binding of Ca^2+^ to purified Chi18H8 was investigated by recording the far-UV CD spectrum during the titration of the apoprotein form of the enzyme (obtained by incubation for 30 min with 20 mM EDTA), with increasing concentrations of CaCl_2_ (5, 10, 100, 1000 and 10,000 μM). The change in CD signals at 219 nm versus CaCl_2_ concentration were fitted to a classical 1:1 complex saturation equation to calculate the dissociation constant, K_d_.

### Determination of mode of action

A chitin-like substrate, i.e. a water-soluble high molecular weight chitosan with a degree of acetylation of 63%, was used as substrate for Chi18H8 to determine the enzyme’s mode of action as previously described [36]. The chitosan (30 mg) was dissolved in 3 mL of 80 mM sodium acetate, pH 5.5. To start the depolymerization reaction, 2.5, 7.5 or 26 µL of Chi18H8 (1.16 mg/mL) were added to 1 mL substrate. After incubating the mixture at 37 °C for 30 min, the reaction was stopped by adjusting the pH to 2.0 and boiling for 10 min. The reaction mixture containing the oligomers was injected and separated on three XK 26 columns in series, packed with Superdex™ 30 (Amersham Pharmacia Biotech, Piscataway, USA) using a refractive index (RI) detector (Shimadzu Schweiz GmbH, Reinach, Switzerland), as previously described [36].

### Antifungal activity assay

Chi18H8 antifungal activity was evaluated by growth repression assays, in triplicate, on *Fusarium graminearum* ATCC 46779, *Rhizoctonia solani* ATCC 10183 and *Botrytis cinerea* ATCC 26943. Plate assays were carried out by centrally inoculating an agar plug, harboring an actively growing fungal culture, in 9-cm diameter Petri plates containing diluted potato dextrose agar (PDA). Sterilized paper discs (Munktell Filter ab, Falun, Sweden) 5 mm in diameter were soaked in 1000, 500, 100 or 10 µg/mL, respectively, of the solubilized Chi18H8 (in 100 mM sodium acetate, pH 5.0) and placed 25 mm around the fungal plug. A liquid assay system was also used. Wells (2 mL cellplate, Biofluidfocus, Largo, USA) containing fungal PDA agar plugs and 200 μL potato dextrose broth (PDB) were added with Chi18H8 (in 100 mM sodium acetate, pH 5.0) to a concentration of 1000, 100 or 10 µg/mL. Boiled enzyme in the same buffer was used as negative control in both assays. The systems were incubated at 23 °C in darkness for 48 h, and longer for *F. graminearum* due to slower mycelial growth rate. Growth of fungal hyphae was monitored daily using a microscope. For the liquid assay, dry weight biomass of the fungal plugs was measured seven days after inoculation.

## Results

### Heterologous expression of the metagenome-sourced Chi18H8

The complete *chi18H8* gene sequence (G + C ratio 64.4%) was previously identified by genetic and expression screening of a high-molecular-weight DNA metagenomic library constructed from a soil in Uppsala (Sweden), characterized as suppressive to club-root disease of cabbage [6, 7, 29]. The gene, consisting of 1275 nucleotides and encoding a protein of 424 amino acids (predicted molecular mass of 45.96 kDa and isoelectric point of 7.75), was obtained by primer walking technique of a fosmid clone [7]. For this work, it was cloned into the pET24b(+) expression plasmid in *E. coli* BL21 Star™(DE3) cells, under the control of the IPTG-inducible *T7* promoter.

**Fig. 1:**
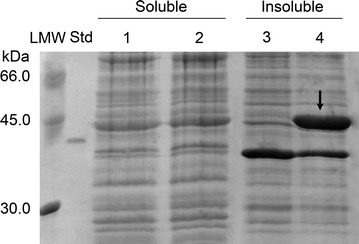
SDS-PAGE analysis of proteins from recombinant *E. coli* BL21 Star™(DE3) cells. *E. coli* BL21 Star™(DE3)/pET24b(+) cells: soluble (*lane 1*) and insoluble (*lane 3*) fractions; *E. coli* BL21 Star™(DE3)/pET24b(+)::*chi18H8* cells: soluble (*lane 2*) and insoluble (*lane 4*) fractions. In each lane, samples corresponding to 0.5 mL of cell culture were loaded. *Std* reference protein, His_6_-GO (2.5 μg, 42.66 kDa), *LMW* standard proteins. Cells were grown in LB medium, protein expression was induced at the early exponential phase by adding 0.4 mM IPTG, and cells were harvested after 24 h incubation at 37 °C. Chi18H8 protein spot is indicated by an *arrow*

Recombinant *E. coli* cells harboring pET24b(+)::*chi18H8* and the control strain carrying the empty vector were grown at the following conditions: (1) five different growth media (LB, TB, SB, autoinduction media 1 and 2); (2) adding 0.4 mM IPTG at the early or at the late exponential phase of growth; (3) keeping temperature after induction at 37 °C or reducing it to 25 or 20 °C; (4) growing cells after induction for different time intervals before harvesting. In all the above experimental conditions, an intense protein band at the expected molecular mass for Chi18H8 was observed in SDS-PAGE only in the insoluble fractions from the recombinant strain harboring the *chi18H8* gene (no signal was detected in cytoplasmic soluble fractions, nor in cell-free culture broths after protein precipitation with trichloroacetic acid). Figure 1 shows the SDS-PAGE when (a) cells were grown in LB, (b) protein expression was induced in the early exponential phase, (c) and temperature was maintained at 37 °C after induction. Similar results were achieved in SB and TB media; lower protein production was observed using the two autoinduction broths [31] or by inducing cells during the late exponential phase of growth (data not shown).

**Fig. 2:**
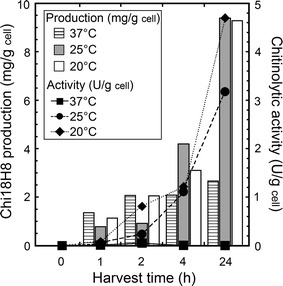
Chi18H8 expression levels in insoluble fractions from *E. coli* BL21 Star™(DE3)/pET24b(+)::*chi18H8* grown in LB medium. Protein expression was induced at the early exponential phase of growth by adding 0.4 mM IPTG, cells were then incubated at 37, 25 or 20 °C, respectively, and harvested after 0, 1, 2, 4 or 24 h. Chi18H8 production (mg/g cell) was determined by densitometric analysis of SDS-PAGE; chitinase activity (U/g cell) was measured fluorimetrically using 4-MU-(GlcNAc)_2_ as a substrate. The values represent the mean of three experiments. Standard error never exceeded 5%

As shown in Fig. 2, accumulation of Chi18H8 into the insoluble fraction increased by prolonging the incubation time after induction, reaching 2.7 mg/g cell (corresponding to 32 mg/L culture in LB medium) after 24 h at 37 °C. Decreasing the incubation temperature to 25 or 20 °C favored the accumulation of recombinant protein into insoluble fractions (more than 9 mg/g cell, corresponding to 55 mg/L culture after 24 h incubation in LB at 20 °C). Fluorimetric enzyme assay of these insoluble fractions revealed that no chitinolytic activity was detectable from cells grown at 37 °C, whereas a weak enzyme activity appeared when growth temperature decreased to 25 and 20 °C (Fig. 2).

Taking all together, these results suggest that Chi18H8 accumulated at 37 °C largely into IBs in an inactive and insoluble form, as often occurs when *E. coli* is used to express metagenomics-sourced enzymes [7, 8]. Indeed, decreasing the expression temperature favored the formation of so-called non-classical IBs, i.e. those IBs that are, at least in part, biologically active since they contain an increasing portion of recombinant active protein molecules [37–39].

### Solubilization of IBs and purification of Chi18H8

Considering the above results and those previously reported [7] on *E. coli* pGEX-6P-3::*chi18H8* producing cytoplasmic Chi18H8 at an extremely low yield, a new approach was tested to recover the chitinase at a higher yield by solubilization of IBs produced by *E. coli* BL21 Star™(DE3)/pET24b(+)::*chi18H8* cells grown in LB and incubated for 24 h at 20 °C after adding IPTG. These IBs, that likely contain some protein molecules in native-like active conformation, were recovered by centrifugation from cell lysate after mild sonication conditions. See Additional file 1: Table S1 for the different solubilization methods that were tested. Briefly, first we tried classical methods, re-suspending IBs in solutions containing strong denaturing agents and chaotropes, such as urea, guanidium hydrochloride (GdnHCl) or guanidium thiocyanate (GdnTC), alone or combined with the detergent Triton X-100, the reducing agent DTT, the chelating agent EDTA or NaCl salt. By increasing the denaturing agent concentration, an increased protein solubilization (up to 85–95%) was observed through SDS-PAGE. However, during the following refolding steps (removing the denaturant agents by dialysis), Chi18H8 aggregation and precipitation occurred and consequently the protein samples were biologically inactive (as checked by the fluorimetric enzyme activity assay). Milder solubilization conditions of IBs (Additional file 1: Table S1) were then evaluated for better preserving the inferred existing native-like secondary structure and reducing the risk of protein aggregation during the purification process. These mild solubilization protocols were based on IB incubation with detergents (Triton X-100, NLS and CHAPS [3-[(3-cholamidopropyl)dimethylammonium]-1-propanesulfonate]), or alkyl alcohol (*n*-propanol), or acid pH solutions (HCl, lactic acid, formic acid). Using the protocols based on detergents and *n*-propanol, the solubilization yield was very low (ca. 5–10%, Additional file 1: Table S1). Recovery of active Chi18H8 was only achieved in the case of IB solubilization in aqueous acid solutions of hydrochloric or lactic acid (but not in formic acid). As reported in Additional file 1: Table S1, this protocol of acid solubilization involved an osmotic shock pre-treatment of cell pellet with 25% (w/v) sucrose for disrupting the outer membrane of *E. coli* cells and removing periplasmic proteins. Additionally, by introducing different and sequential washing steps (with low concentrations of detergents like DOC, Triton X-100 and Nonidet P-40) after sonication of the IBs and prior to protein solubilization, IB purity increased and contaminants were reduced during the following steps of Chi18H8 purification; actually the washing steps contributed to the removal of membrane fragments, see [37, 40].

**Fig. 3:**
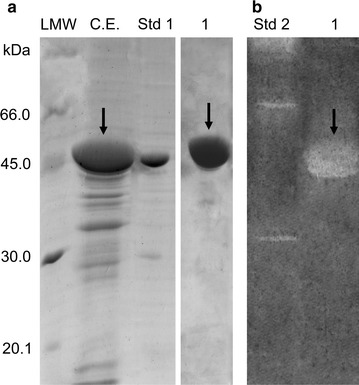
**a** SDS-PAGE and **b** zymogram analysis of Chi18H8 purification. *C.E*. crude extract, raw enzyme after solubilization from IBs in 10 mM lactic acid and dialysis against 100 mM HEPES pH 5.6 (loaded onto WA11 resin). *1* fraction corresponding to the flow-through. *Std 1* reference protein, His_6_-GO (5 μg, 42.66 kDa). *Std 2* zymogram reference protein, chitinases from *Trichoderma viride* (Sigma-Aldrich). *LMW* standards proteins. Chi18H8 protein spots are indicated by *arrows*

Thus, by re-suspending IBs in 10 mM hydrochloric or lactic acid we achieved solubilization yields of more than 90 or 80%, respectively, as shown in Table 1. However, in the case of IB solubilization in 10 mM lactic acid, the specific activity of the recovered Chi18H8 (40.7 U/mg protein) was definitely higher than in hydrochloric acid (29.6 U/mg protein). Increasing the acid concentration to 100 mM led to a reduction of both solubilization yield and specific activity. SDS-PAGE analysis revealed that the solubilized Chi18H8 in 10 mM lactic acid was 70% pure (Fig. 3a; Table 2).

**Table 1:** Chi18H8 solubilization from IBs of *E. coli* BL21 Star™(DE3)/pET24b(+)::*chi18H8* cells in acidic pH conditions

Solubilization agent	Concentration (mM)	Solubilization yield (%)	Specific activity (U/mg_Chi18H8_)^a^
HCl	10	93 ± 4.0	29.6 ± 1.2
	100	20 ± 2.0	15.9 ± 0.9
Lactic acid	10	82 ± 3.5	40.7 ± 1.4
	100	60 ± 6.5	30.7 ± 1.3

Solubilization yield was estimated by densitometric analysis of proteins separated by SDS-PAGE. Enzyme activity was measured by fluorimetric assay on 4-MU-(GlcNAc)_2_

^a^Values represent the mean of three independent experiments (mean ± standard error)

**Table 2:** Purification of Chi18H8 from IBs of *E. coli* BL21 Star™(DE3)/pET24b(+)::*chi18H8* cells

	Volume	Activity	Protein concentration	Specific activity	Purification	Recovery	
	(mL)	(U tot)	(mg/mL)	(U/mg_protein_)	(fold)	(%)	(mg/g_cell_)
IBs	2.0	4.7	11.2	0.2	1.0	100.0	9.4
Soluble fraction from IBs	10.0	314.0	1.1	28.5	66.8	82.0	7.7
Purified Chi18H8	25.3	355.7	0.2	63.9	75.7	57.4	5.4

Enzymatic activity was measured by fluorimetric assay on 4-MU-(GlcNAc)_2_. Protein concentration was determined by the Biuret assay

In order to further purify Chi18H8, different precipitation methods and chromatographic procedures were then evaluated. Salts, including NaCl, KCl, MgCl_2_ and NH_4_(SO_4_)_2_, were added to obtain a selective Chi18H8 precipitation, so were increasing concentrations of ethanol and the addition of CM-chitin-RBV. However, the enzyme co-precipitated with most of the contaminating proteins (data not shown). Different chromatographic methods were tested using sixteen resins, as listed in Additional file 1: Table S2. At most of the conditions, Chi18H8 did not interact with the resins and the chitinolytic activity was mainly found in the flow-through fractions, together with most of the contaminating proteins. In some cases the protein precipitated and, in general, it was inactive if the pH increased above 7.0. The weak cationic exchanger P11 and the hydrophobic resin HP20SS retained Chi18H8; however, from neither resin the enzyme could be eluted in a biologically active form (Additional file 1: Table S2). Interestingly, when the weak anionic exchanger WA11 resin was used at HIC conditions, most of the contaminating proteins were retained by the resin, allowing a partial purification of the chitinase from the flow-through in 100 mM HEPES pH 5.6. In this case, the purified protein was >95% pure (Fig. 3a) and the zymogram analysis in semi-denaturing conditions with CM-chitin-RBV confirmed the chitinolytic activity of the purified enzyme (Fig. 3b). Indeed, none chitinolytic activity was found when the contaminating proteins were eluted from WA11 resin by an isocratic elution using 50% (v/v) ethanol in 100 mM HEPES pH 5.6. As summarized in Table 2, the two-step lactic acid solubilization/HIC purification method allowed the recovery of more than 55% of the recombinant enzyme accumulated into IBs, with a 320-fold increase of specific activity (from 0.2 U/mg protein in the IBs, to 63.9 U/mg protein after HIC purification).

### Chi18H8 production in 3 L bench- and 30 L industrial-bioreactors

**Fig. 4:**
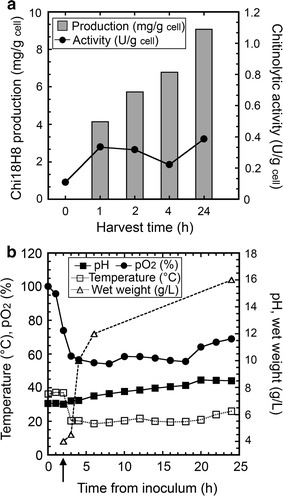
Chi18H8 production at 3 L bench-bioreactor scale. **a** Chi18H8 production (mg/g cell) into insoluble fractions was determined by densitometric analysis of proteins separated through SDS-PAGE. Chitinolytic activity was measured by fluorimetric assay on 4-MU-(GlcNAc)_2_. **b** Time course of pH, pO_2_, temperature and wet weight. Induction of protein expression with 0.4 mM IPTG is indicated by an *arrow*. The values represent the mean of three experiments. Standard error never exceeded 10%

Chi18H8 production and purification was scaled-up first in 3 L and then in 30 L bioreactors, where *E. coli* BL21 Star™(DE3)/pET24b(+)::*chi18H8* cells were grown at the conditions optimized for flask fermentation. Figure 4 shows the time course of cell growth and Chi18H8 production in the 3 L bench-fermenter. Heterologous protein production was induced by adding IPTG to cells in the early stationary phase of growth. The maximum chitinase production in IBs after 24 h was 9.1 mg/g cell (corresponding to 75 mg/L) and 12.9 mg/g cell (equal to 80 mg/L) for the 3 L and the 30 L bioreactor, respectively. These amounts were comparable or even higher than what was obtained at flask level (Table 3).

**Table 3:** Comparison of Chi18H8 production and IB solubilization in 2 L flasks, 3 L bench-bioreactor or 30 L industrial-bioreactor

	Cell weight (g_cell_/L)	Chi18H8 production (mg/g_cell_)	Solubilization yield (%)	Specific activity (U/mg_protein_)
Flask	4.0	9.4	>80	40.7
3 L bench-bioreactor	5.3	9.1	>65	52.1
30 L industrial-bioreactor	6.2	12.9	>65	48.4

Protein expression was induced in the early exponential growth phase (OD_600nm_ ~0.6) with 0.4 mM IPTG and cells were harvested after 24 h of incubation. Chi18H8 production (mg protein/g cell) and solubilization yield in 10 mM lactic acid were estimated by densitometric analysis of proteins separated by SDS-PAGE. Specific activity of the solubilized enzyme was measured by the fluorimetric assay on 4-MU-(GlcNAc)_2_

The following IB solubilization in 10 mM lactic acid allowed, from cells grown at both reactor sizes, the recovery of active Chi18H8 with a >65% yield and a specific activity of 52.1 and 48.4 U/mg protein, respectively (Table 3). Solubilized protein was about 70% pure, as revealed by SDS-PAGE densitometric analysis, similarly to the results obtained with the protein produced in flasks (not shown). Also purification using HIC with the weak anionic exchanger WA11 resin turned out to be scalable and suitable for the production of Chi18H8. A volume of 135 mL solubilized Chi18H8 in 100 mM HEPES pH 5.6, corresponding to 2.2 L of culture, was loaded onto a column of WA11 resin (40 mL). Also in this case, the Chi18H8 protein was found in the flow-through, whereas contaminating proteins were retained by the resin. Chi18H8 recovery yield was approximately 70% and the purity was above 95%.

### Chi18H8 characterization

#### Substrate specificity

Using the fluorimetric assay on three different-length analogues of natural chitooligosaccharides, pure Chi18H8 solubilized from IBs in lactic acid showed a prevalent chitobiosidase activity (40.7 U/mg), a weaker endochitinase activity (7.5 U/mg) and no β-*N*-acetyl-glucosaminidase activity, confirming what reported for the recombinant enzyme previously isolated [7]. Additionally, the enzyme was able to hydrolyze colloidal chitin, with an estimated activity of 0.41 U/mg.

#### Mode of action

**Fig. 5:**
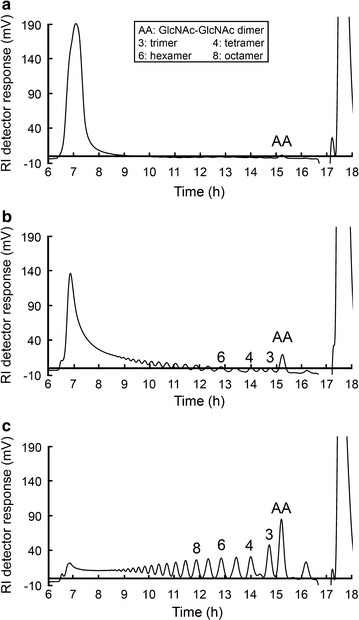
Size distribution of chitosan oligomers after Chi18H8 degradation of a water-soluble chitin-like substrate (chitosan with a 63% degree of acetylation). The chromatograms (using a refractive-index detector) show the size-distribution of the oligomers upon incubating the chitosan substrate (10 mg) with increasing amounts of Chi18H8 (1.16 mg/mL): 2.5 μL (**a**), 7.5 μL (**b**), and 26 μL (**c**). Peaks are labelled with the length of the oligomer or the sequence of the oligomer [AA is the chitin dimer (GlcNAc–GlcNAc)], and were identified by comparison with standard chitin/chitosan oligomers

Size-exclusion chromatograms revealing the length distribution of the COs when increasing amounts of Chi18H8 were added to a water-soluble chitin-like substrate, i.e. a high-molecular weight chitosan with a degree of acetylation of 63%, are shown in Fig. 5. The molecules eluting first in the chromatograms (after 7 h) are the largest molecules (e.g. chitosan with more than 50 units). The signal at the other end of the chromatograms (18 h) is the salt peak. Furthermore, the chitin dimer (GlcNAc–GlcNAc, labelled AA in Fig. 5) is the peak eluting between 15 and 15.5 h, the peaks eluting at 14 and 13.4 h are respectively the tetramer (labelled 4 in the figure) and the pentamer, and so on [36]. With increasing amounts of Chi18H8 added to the substrate, the chromatograms show that the chitosan peaks (eluting at 7 h) are gradually reduced and a continuum of oligomers appears as a result of the hydrolysis of the polymeric chitosan chain. The continuum of COs in the chromatograms with no preference for even-numbered oligomers (Fig. 5) indicates that Chi18H8 operates according to an endo-mode of action [36].

#### pH and temperature profile

**Fig. 6:**
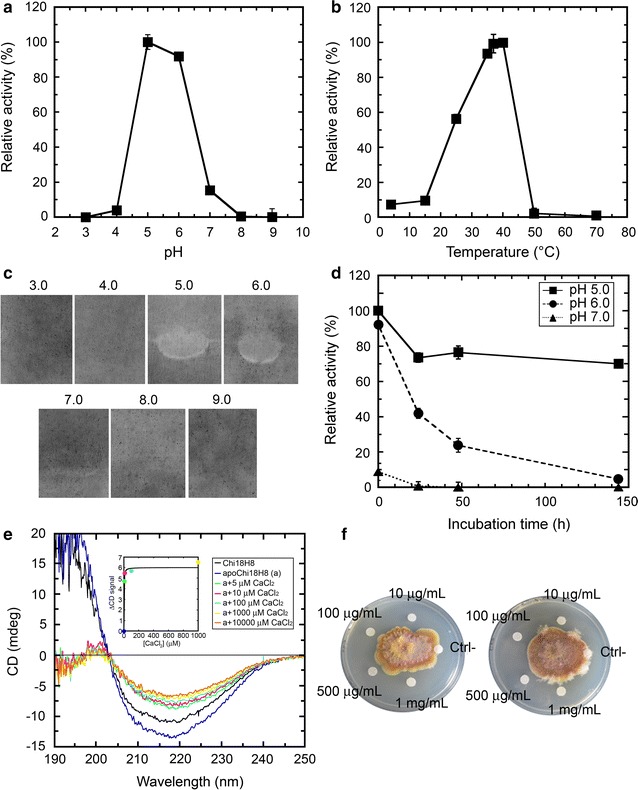
Characterization of Chi18H8 purified from IBs. **a** pH profile of Chi18H8 activity, using 4-MU-(GlcNAc)_2_ as substrate. **b** Temperature influence of Chi18H8 activity on 4-MU-(GlcNAc)_2_ measured in 100 mM sodium acetate buffer, pH 5.0. **c** Effect of pH on Chi18H8 activity on CM-chitin-RBV (zymograms in semi-denaturing conditions). **d** Time course of stability of Chi18H8 at different pHs. The enzyme was incubated in 100 mM sodium acetate pH 5.0, sodium phosphate pH 6.0 or sodium phosphate pH 7.0 for up to 144 h at 30 °C. At various time intervals, samples were withdrawn and the residual chitinolytic activity was fluorimetrically measured on 4-MU-(GlcNAc)_2_ under standard conditions. In panels **a**, **b** and **d**, the activity is expressed as a percentage of the maximum activity (set as 100%) and the values represent the mean of three experiments (mean ± standard error). **e** Circular dichroism (CD) far-UV spectra of the purified chitinase (indicated as ‘Chi18H8’), of the same preparation following EDTA addition and dialysis to eliminate divalent ions (‘apoChi18H8’), and after adding increasing concentrations of CaCl_2_. The concentration of Chi18H8 was 0.2 mg/mL in 10 mM HEPES pH 5.6. In the *inset*, the ΔCD signals at 219 nm vs. CaCl_2_ concentration used to extrapolate the dissociation constant K_d_, are reported. **f** Antifungal activity of Chi18H8 repressing *Fusarium graminearum* growth on PDA plates. Reduction in radial growth of the centrally inoculated fungi was evident around discs embedded of Chi18H8 at 1000, 500, 100 or 10 µg/mL concentration. No growth inhibition was observed in the negative control (Ctrl^−^), i.e. boiled Chi18H8. The image shows two replicates (out of three)

The optimum pH for the chitinolytic activity of the purified enzyme was between 5.0 and 6.0 and only 15.3% of the activity was maintained at pH 7.0, whereas no activity was detected at pH 3.0 and 4.0 and above pH 8.0 (Fig. 6a). Consistently, the chitinase was able to hydrolyze the CM-chitin-RBV only in the 5.0–7.0 pH range, and no activity was detectable in zymograms at pH 3.0, 4.0, 8.0 or 9.0 (Fig. 6c). The optimum temperature for chitobiosidase activity on 4-MU-(GlcNAc)_2_ was between 35 and 40 °C. More than 55% of the activity was retained at 25 °C (Fig. 6b); at 50 °C the enzyme was inactive. The stability of Chi18H8 was investigated by measuring the residual activity following 144 h of incubation in buffers at different pHs (5.0, 6.0 and 7.0) at the temperature of 30 °C. When Chi18H8 was incubated at pH 7.0, a complete loss of activity was observed within 24 h. At pH 6.0, the activity dramatically decreased to 5% of the initial activity within 144 h. At pH 5.0, however, Chi18H8 retained about 70% of its initial activity after 144 h (Fig. 6d).

#### Effect of metal ions, detergents, salts and other compounds

The effect of several compounds was tested on Chi18H8 enzyme activity (Table 4). The metal ions Cu^2+^, Fe^2+^, Mg^2+^, Mn^2+^, Ni^2+^, Zn^2+^, as well as the monovalent cation NH_4_^+^, reduced the hydrolytic activity of Chi18H8. The strongest inhibition was caused by Cu^2+^ and Fe^2+^. On the contrary, Ca^2+^, K^+^ and Co^2+^ slightly increased the enzyme activity. The chelating agent EDTA gave an inhibitory effect, thus suggesting that Chi18H8 activity could be promoted by the presence of specific metal ions, in particular Ca^2+^ ions (see below).

**Table 4:** Effect of different compounds on Chi18H8 activity

Compounds	Final concentration	Relative activity (%)
Control		100.0
Metal ions	mM	
Ca^2+^	20	126.8 ± 0.8
Cu^2+^	20	0.1
Fe^2+^	20	0.1
K^+^	20	124.2 ± 3.1
Mg^2+^	20	86.4 ± 1.4
Mn^2+^	20	49.9 ± 0.1
Ni^2+^	20	64.3 ± 0.1
NH_4_^+^	20	68.0 ± 7.7
Zn^2+^	20	16.6 ± 2.0
Co^2+^	20	119.6 ± 1.5
Chelating agents:	mM	
EDTA	20	53.6 ± 0.1
Reducing agents	mM	
2-mercaptoethanol	20	1.1 ± 2.1
DTT	10	20.5 ± 6.7
Detergents	% (w/v)	
SDS	1	0.0
Triton X-100	1	72.2 ± 2.5
Tween-20	1	24.0 ± 3.6
DOC	1	120.4 ± 0.1
Nonidet P-40	1	91.0 ± 2.0
NLS	1	0.0
Sugars	mM	
GlcNAc	10	104.1 ± 3.3
Chitobiose	10	82.0 ± 1.9
Organic solvents:	% (v/v)	
Ethanol	10	90.9 ± 2.5
Methanol	10	88.6 ± 2.3
* n*-Propanol	10	69.4 ± 1.9
DMSO	10	97.7 ± 14.4
Salt	M	
NaCl	0.1	38.5 ± 1.8
NaCl	0.25	29.9 ± 8.1
NaCl	0.5	16.6 ± 0.1
NaCl	1	8.6 ± 0.4
NaCl	2	4.4 ± 2.5

The activity was measured by the fluorimetric assay on 4-MU-(GlcNAc)_2_. Activity values are reported as relative to the value of 40.7 U/mg protein (set as 100%) recorded for purified Chi18H8 at 37 °C in 100 mM sodium acetate, pH 5.0. Values represent the means of three independent experiments (mean ± standard error)

The reducing agents 2-mercaptoethanol and DTT strongly reduced the chitinase activity. Among the detergents, SDS, NLS, Tween-20 and Triton X-100 abolished or reduced the chitinase activity. Nonidet P-40 showed only a slight inhibitory effect on enzyme activity, whereas DOC stimulated the activity. Chi18H8 activity was only slightly affected in the presence of organic solvents (10% v/v final concentration). The presence of the monosaccharide GlcNAc did not influence the activity of Chi18H8, whereas 10 mM chitobiose was slightly inhibitory. High salt concentrations, as shown in Table 4, inhibited the chitinase, which retained only 4.4% of its activity when incubated with 2 M NaCl.

#### Secondary structure analysis

CD spectrum of the purified chitinase in the far-UV region (Fig. 6e, in 10 mM HEPES buffer pH 5.4) indicates a prevalence of β-sheets (~36.6%) and ~2.2% of α-helices. Following the incubation with 20 mM EDTA and dialysis, the spectrum of the enzyme was slightly changed, showing a more negative value of the peak at 219 nm, thus pointing to the generation of a metal-free enzyme form (i.e. the apoprotein, Fig. 6e). Notably, a significant alteration in far-UV CD spectrum was evident adding CaCl_2_ to the apoprotein of Chi18H8 (Fig. 6e). From the plot of ΔCD signal at 219 nm vs. CaCl_2_ concentration (inset in Fig. 6e), a K_d_ = 1.27 ± 0.33 μM was estimated, indicating a strong interaction of Ca^2+^-ions with the apoprotein moiety. The addition of Zn^2+^ also altered Chi18H8 CD spectrum similarly to Ca^2+^-ions (not shown) although Zn^2+^-ions were inhibitors of the enzymatic activity (see Table 4).

#### Antiphytopathogen activity

Chi18H8 was active against the phytopathogen fungus *F. graminearum* (Fig. 6f) and to a less extent against *R. solani* (Additional file 2: Figure S1). Reduction in radial growth of *F. graminearum* on PDA plates was evident around discs with purified Chi18H8 at concentrations of 1000, 500, 100 or 10 μg/mL (Fig. 6f). No growth inhibition was observed in the negative control (boiled enzyme). PDA plate assays with *R. solani* did not reveal a so clear growth inhibition effect as with *F. graminearum*, whereas no growth inhibition at all was observed in the case of the fast growing *Botrytis cinerea* (not shown). Liquid assays inoculated with *F. graminearum* and *R. solani* (Additional file 2: Figure S1), confirmed that growth of both fungi was reduced (even if at different extent) in the presence of Chi18H8, as measured as lower dry weight after incubation compared with control (boiled enzyme).

## Discussion

Functional metagenomics is a powerful tool for accessing the genetic diversity encrypted in the environmental microbial communities, hence discovering novel enzymes useful for biotechnological applications, for example the broadly applicable chitinolytic enzymes [6–8, 41]. However, for any downstream industrial application, high-level production of the candidate enzyme for sustaining its chemical and biological characterization is central. Consequently, the development of suitable expression systems for the over-expression of proteins, together with the identification of efficient recovery and purification protocols, still represent the major bottlenecks for the biotechnological exploitation of microbial community’s genomes through the application of metagenomics.

*Escherichia coli* typically remains the first-choice host for library construction and recombinant protein production due to the convenience in cloning procedure, low cultivation costs, rapid growth and high expression level of many heterologous proteins. Unfortunately, one intrinsic limit of *E. coli* as a heterologous host for functional metagenomics depends on its poor secretory machinery; to limit toxicity and metabolic burden, *E. coli* tends to accumulate heterologous proteins into IBs. Consequently, *E. coli* is predicted to readily express no more than approximately 40% of the heterologous genes into biologically active proteins [42].

In this study, we developed a protein-tailored production and purification process for the novel metagenome-sourced chitinase Chi18H8 based on mild solubilization of recombinant *E. coli* IBs. Recombinant *E. coli* strains (*E. coli* pGEX-6P-3::*chi18H8* used in [7] and *E. coli* pET24b(+)::*chi18H8* used in this work) massively accumulated Chi18H8 into IBs. Notably, in the latter strain, a marginal chitinolytic activity was detectable when the temperature of growth was decreased from 37 to 20 °C after adding IPTG. As mentioned in the Results, biologically active IBs may be considered as non-classical IBs, presumably containing a loose arrangement of recombinant protein molecules co-existing in unfolded, partially folded or even native active structures [37, 38, 43, 44]. In the last decade, advanced structural techniques indicated that most IBs possess conformational heterogeneity, with amyloid structure building a network in which protein molecules with various conformations, including native, are trapped [45]. Consistently, we found that it was advantageous to adopt mild solubilization methods to recover Chi18H8 from partially active IBs, since these methods might preserve the existing native-like secondary structure of proteins, reducing the risk of protein re-aggregation during the refolding step [37, 46]. The protocol of mild solubilization that was developed for Chi18H8—without using denaturing and chaotropic agents—was based on a few IB washing steps, followed by freezing and thawing, and re-suspension in lactic acid. Solubilization of *E. coli* IBs using hydrochloric acid was introduced by Okumura et al. [47] for recovering the cytotoxic protein Cry45Aa of *Bacillus thuringiensis*. To our knowledge, our work is the first reporting on the use of lactic acid for IB solubilization.

An advantage of IBs is that these are specifically concentrated aggregates of the recombinant protein of interest that can be easily recovered from the cell *milieu*. In order to obtain a highly pure (≈95%) Chi18H8, IB solubilization in lactic acid was coupled to a further purification step based on hydrophobic-interaction chromatography, commonly employed for industrial purification of peptides and proteins [48]. The use of the weak anionic exchanger WA11 in a “negative mode” (i.e. retaining all the contaminating proteins on the resin) allowed the enzyme purification. Importantly, both upstream (cultivation and protein expression induction) and downstream (IB recovery and solubilization, and protein purification) processes were scalable from shaken flasks to 3 L bench- and to 30 L industrial pilot scale-bioreactors, giving comparable results in terms of recombinant *E. coli* biomass, IB solubilization and purification yields, and Chi18H8 biological activity. The process could be considered robust, reproducible and high-yielding: ca. 37 mg/L of pure active protein was obtained at the pilot scale of 30 L fermenter. This is the highest purification yield thus far obtained for a metagenome-sourced chitinase: actually, the two other chitinolytic enzymes identified by metagenomics and expressed in *E. coli* were recovered with a yield of ca. 0.6 mg/L for 53D1 [8] and ca. 8.3 mg/L for ChiA01 [26]. Notably, Chi18H8 purification yield also favorably compared with those obtained for other recombinant chitinolytic enzymes produced in *E. coli*: only few micrograms per liter of culture were obtained in the case of *Bacillus circulans* WL-12 ChiA (95 μg/L) [49], whereas milligrams of protein were prepared for HschiA1 from *Halobacterium salinarum* CECT 395 (1.4 mg/L) [50], for ChiCH and ChiCW from *Bacillus cereus* (4.6 and 2.3 mg/L, respectively) [51], for *Bacillus licheniformis* ChiA (20 mg/L) [52] and for *Paecilomyces thermophila* PtChiA (28 mg/L) [53].

Once Chi18H8 was purified, its prevalent chitobiosidase activity [7] was confirmed (specific activity of 40.7 U/mg on 4-MU-(GlcNAc)_2_): the pure enzyme was active not only on synthetic chitooligosaccharides used in the fluorimetric assay, but also on the complex substrate colloidal chitin (0.4 U/mg). Chi18H8 was found to operate according to a non-processive endomode of action on a water-soluble chitin-like substrate, in a similar mode of action as ChiC from *Serratia marcescens*, that, among hundreds of different microorganisms tested for chitinolytic activities, remains the most efficient one [54, 55]. Specific activity of pure Chi18H8 on fluorigenic analogues of chitooligosaccharides is within the range reported for other bacterial chitinases belonging to the 18GH family and for the commercial chitinolytic enzyme cocktail from the fungus *Trichoderma viride* (in this last case specific activity was of 35.9, 18.1 and 12.1 U/mg on 4-MU-GlcNAc, 4-MU-(GlcNAc)_2_ and 4-MU-(GlcNAc)_3_, respectively) [11]. We recently reported that the metagenome-sourced 53D1 showed a specific activity of 45.2 U/mg on 4-MU-(GlcNAc)_2_ and of 21.2 U/mg on 4-MU-(GlcNAc)_3_ [8]. The activities of PbChi70 from *Paenicibacillus barengoltzii* [56] and ChiA74 from *B. thuringiensis* [57] on 4-MU-(GlcNAc)_3_ were 13.5 and 75.2 U/mg, respectively. Indeed, Chi18H8 is less active on colloidal chitin if compared with other 18GH family-chitinases, such as 53D1 (2.3 U/mg) [8], *B. licheniformis* ChiA (1.5 U/mg) [52], *Halobacterium salinarum* CECT 395 HschiA1 (16.5 U/mg) [50], and *Paenicibacillus berengoltzii* PbChi70 (30.1 U/mg) [56].

Chi18H8 is a mesophilic enzyme, active and stable in acid pH conditions, consistently with the characteristics of the soil from which it was isolated [6, 7, 29]. Characterization suggests that Chi18H8 activity is stimulated by Ca^2+^ binding: its activity was significantly reduced in the presence of EDTA, possibly because of chelation of calcium ions necessary for correct protein structure and the catalytic site, as shown for some other chitinases [50, 58]. In accordance, the presence of calcium ions increased Chi18H8 activity and altered the protein secondary structure, according to circular dichroism analysis. Chi18H8 activity was strongly inhibited by sodium chloride, iron and copper and, to a minor extent, by manganese and nickel ions, as in the case of several other chitinolytic enzymes [59, 60]. A particularly relevant property of Chi18H8 for biotechnological application is the high solvent tolerance, indicating that it could be tested in non-aqueous solutions. Finally, growth inhibition assays conducted with the pure enzyme confirmed Chi18H8 antifungal activity on the important plant pathogen *F. graminearum* [61] and, to a less extent, on *R. solani* [62].

In conclusion, Chi18H8 shows interesting features including activity on complex chitin substrates, antifungal activity and stability at conditions compatible with possible *in field* applications, e.g. its range of activity is adequate for inhibiting phytopathogen fungal growth typically occurring in acidic and mesophilic environments. Additionally, this work contributes to expanding the methods for efficiently recovering active proteins by mild solubilization of *E. coli* IBs using lactic acid and coupling it with a single step purification by hydrophobic interaction chromatography.

## Additional files

**Additional file 1.** Evaluation of Chi18H8 antifungal activity in liquid assays. **Figure S1**. Growth in liquid assays of *Fusarium graminearum* ATCC 46779 and *Rhizoctonia solani* ATCC 10183 in the presence of increasing concentrations of Chi18H8.

**Additional file 2.** Screening of methods for recovery and solubilization of Chi18H8 from *Escherichia coli* inclusion bodies (IBs) and its purification. **Table S1**. Different protocols used for isolation and solubilization of IBs and, eventually, for refolding Chi18H8. **Table S2**. Affinity chromatography (AC), ion exchange chromatography (IEC) and hydrophobic interaction chromatography (HIC) pilot experiments for Chi18H8 purification.

## Abbreviations

CD: circular dichroism
CEs: chitinolytic enzymes
CHAPS: 3-[(3-cholamidopropyl)dimethylammonium]-1-propanesulfonate
CM-chitin-RBV: carboxymethyl-chitin-remazol-brilliant-violet
COs: chitooligosaccharides
DMSO: dimethyl sulfoxide
DNase: deoxyribonuclease
DOC: sodium deoxycholate
DTT: dithiothreitol
EDTA: ethylenediaminetetraacetic acid
GdnHCl: guanidium hydrochloride
GdnTC: guanidium thiocyanate
GH: glycosyl hydrolase
GlcNAc: *N*-acetylglucosamine
GO: glycine oxidase
HEPES: 4-(2-hydroxyethyl)-1-piperazineethanesulfonic acid
HIC: hydrophobic interaction chromatography
IBs: inclusion bodies
IPTG: isopropyl β-d-thiogalactopyranoside
K_d_: dissociation constant
LB: Luria-Bertani broth
4-MU-GlcNAc: 4-methylumbelliferyl *N*-acetyl-β-d-glucosaminide
4-MU-(GlcNAc)_2_: 4-methylumbelliferyl *N*,*N’*-diacetyl-β-d-chitobioside
4-MU-(GlcNAc)_3_: 4-methylumbelliferyl *N,N’,N’’*-triacetyl-β-d-chitotrioside
NaPPi: sodium pyrophosphate
NLS: *N*-lauroylsarcosine
OD: optical density
PBS: phosphate buffer saline
PDA: potato dextrose agar
PDB: potato dextrose broth
PMSF: phenylmethylsulfonylfluoride
RI: refractive index
rpm: revolutions per minute
SB: super broth
SDS: sodium dodecyl sulfate
SDS-PAGE: sodium dodecyl sulfate-polyacrylamide gel electrophoresis
STE: sodium chloride-Tris-EDTA buffer
TB: terrific broth
