# Supplementary material for: Production and characterization of a novel antifungal chitinase identified by functional screening of a suppressive-soil metagenome
Source: Microb Cell Fact. 2017 Jan 31;16:16. doi: 10.1186/s12934-017-0634-8 (PMC5282697; doi:10.1186/s12934-017-0634-8)
Supplement: Supplementary file 1 — Additional file 1. Evaluation of Chi18H8 antifungal activity in liquid assays. Figure S1. Growth in liquid assays of Fusarium graminearum ATCC 46779 and Rhizoctonia solani ATCC 10183 in the presence of increasing concentrations of Chi18H8. [file 12934_2017_634_MOESM1_ESM.docx]

**Supplementary materials. Additional File 1.**

**Screening of methods for recovery and solubilization of Chi18H8 from *Escherichia coli* inclusion bodies (IBs) and its purification**

*E. coli* BL21 Star^TM^(DE3)/pET24b(+)::*chi18H8* cells were grown in LB medium, protein expression was induced in the early exponential phase by adding 0.4 mM IPTG, and after 24 h of incubation at 20°C, cells were harvested by centrifugation at 3220 x *g* for 20 min.

For the sake of clarity, the strategy for purifying Chi18H8 from IBs is divided into four major steps: *(i)* isolation of IBs, *(ii)* solubilization of proteins from IBs, *(iii)* refolding of solubilized Chi18H8 and *(iv)* purification of Chi18H8 by chromatographic techniques. The different methods tempted for isolation and solubilization of IBs, and for refolding the solubilized protein molecules into native conformation, are listed in Table S1, whereas the chromatographic conditions tested for Chi18H8 purification are reported in Table S2.

After washing with sodium chloride-tris-EDTA (STE buffer: 10 mM Tris-HCl pH 8.0, 1 mM EDTA, 100 mM NaCl), when IBs were solubilized by conventional harsh methods (Table S1), cells were suspended in 2 mL/g cell phosphate buffer saline (PBS) pH 7.3 containing 10 µg/mL deoxyribonuclease (DNase), 0.19 mg/mL phenylmethylsulfonylfluoride (PMSF) and 0.7 mg/mL pepstatin, and sonicated on ice (10 cycles of 30 s each, with a 30-s interval, using a Branson Sonifier 250, Danbury, USA). IBs were then recovered by centrifugation at 20000 × *g* for 1 h at 4°C, dissolved in 10 mL/g cell of one of the solubilization buffers listed in Table S1 and incubated for 2 h in weak agitation at 4°C. Insoluble material was removed by centrifuging at 12000 × *g* at 4°C for 10 min. Protein refolding was finally accomplished with three-step dialysis (for dialysis buffer composition and step duration see Table S1).

The same sonication protocol above described was used for IB mild solubilization procedures based on the use of detergents and solvents (Table S1). In these cases, recovered IBs were suspended in 10 mL/g cell of solubilization buffer (Table S1) and incubated for 5 h in vigorous agitation at room temperature, followed by centrifugation at 12000 × *g* at 4°C for 10 min to remove insoluble material.

Alternatively, in the case of solubilization in acid solutions (Table S1), *E. coli* cells were first subjected to osmotic shock (by incubating them for 30 min in 5 mL/g cell of 50 mM Tris-HCl pH 8.0, 25% (w/v) sucrose, 1 mM EDTA [ethylenediaminetetraacetic acid]) and then sonicated on ice for 6 cycles (for details, see also the Methods section). Samples of bioactive Chi18H8 prepared by osmotic shock and solubilization of IBs in 10 mM lactic acid, were dialyzed against the activation buffers listed in Table S2. Different resins and chromatographic conditions were used as reported in Table S2.

| **Table S1** **Different protocols used for isolation and solubilization of IBs and, eventually, for refolding Chi18H8**   \| **IB isolation** \| **IB solubilization**  **(buffer composition)** \| **Solubilization yield** \| **Refolding**  **(dialysis buffer composition and step duration)** \| **Precipitation during dialysis** \| **Solubilized protein activity** \| \| --- \| --- \| --- \| --- \| --- \| --- \| \| **Harsh solubilization methods** \| \| \| \| \| \| \| Sonication \| 10 mM Tris-HCl pH 8.0, 8 M urea, 100 mM NaCl \| 85% \| 1) 10 mM Tris-HCl pH 8.0, 4 M urea, 100 mM NaCl, 1h 45min \| Yes \| No \| \| 2) 10 mM Tris-HCl pH 8.0, 2 M urea, 100 mM NaCl, 1h 45min \| \| 3) 10 mM Tris-HCl pH 8.0, 100 mM NaCl, O.N. \| \| 10 mM Tris-HCl pH 8.0, 2 M urea, 100 mM NaCl, 2 mM EDTA, 0.1% (v/v) Triton X-100 \| < 5% \| 1) 10 mM Tris-HCl pH 8.0, 1 M urea, 100 mM NaCl, 2 mM EDTA, 0.1% (v/v) Triton X-100, 1h 45min \| No \| No \| \| 2) 10 mM Tris-HCl pH 8.0, 0.5 M urea, 100 mM NaCl, 2 mM EDTA, 0.1% (v/v) Triton X-100, 1h 45min \| \| 3) 10 mM Tris-HCl pH 8.0, 100 mM NaCl, 2 mM EDTA, 0.1% (v/v) Triton X-100, O.N. \| \| 10 mM Tris-HCl pH 8.0, 5 M urea, 100 mM NaCl, 2 mM EDTA, 0.1% (v/v) Triton X-100 \| 30% \| 1) 10 mM Tris-HCl pH 8.0, 4 M urea, 100 mM NaCl, 2 mM EDTA, 0.1% (v/v) Triton X-100, 1h 45min \| No \| No \| \| 2) 10 mM Tris-HCl pH 8.0, 2.5 M urea, 100 mM NaCl, 2 mM EDTA, 0.1% (v/v) Triton X-100, 1h 45min \| \| 3) 10 mM Tris-HCl pH 8.0, 100 mM NaCl, 2 mM EDTA, 0.1% (v/v) Triton X-100, O.N. \| \| 10 mM Tris-HCl pH 8.0, 8 M urea, 100 mM NaCl, 2 mM EDTA, 0.1% (v/v) Triton X-100 \| 90% \| 1) 10 mM Tris-HCl pH 8.0, 4 M urea, 100 mM NaCl, 2 mM EDTA, 0.1% (v/v) Triton X-100, 1h 45min \| Yes \| No \| \| 2) 10 mM Tris-HCl pH 8.0, 2 M urea, 100 mM NaCl, 2 mM EDTA, 0.1% (v/v) Triton X-100, 1h 45min \| \| 3) Tris-HCl 10 mM pH 8.0, urea 4 M, NaCl 100 mM, EDTA 2 mM, Triton X-100 0.1% (v/v), O.N. \| \| 10 mM Tris-HCl pH 8.0, 8 M urea, 5 mM NaCl, 2 mM EDTA, 0.1% (v/v) Triton X-100 \| 85% \| 1) 10 mM Tris-HCl pH 8.0, 4 M urea, 5 mM NaCl, 2 mM EDTA, 0.1% (v/v) Triton X-100, 1h 45min \| Yes \| No \| \| 2) 10 mM Tris-HCl pH 8.0, 2 M urea, 5 mM NaCl, 2 mM EDTA, 0.1% (v/v) Triton X-100, 1h 45min \| \| 3) 10 mM Tris-HCl pH 8.0, 5 mM NaCl, 2 mM EDTA, 0.1% (v/v) Triton X-100, O.N. \| \| 10 mM Tris-HCl pH 8.0, 8 M urea, 100 mM NaCl, 1 mM DTT, 0.1% (v/v) Triton X-100 \| 85% \| 1) 10 mM Tris-HCl pH 8.0, 4 M urea, 100 mM NaCl, 1 mM DTT, 0.1% (v/v) Triton X-100, 1h 45min \| Yes \| No \| \| 2) 10 mM Tris-HCl pH 8.0, 2 M urea, 100 mM NaCl, 1 mM DTT, 0.1% (v/v) Triton X-100, 1h 45min \| \| 3) 10 mM Tris-HCl pH 8.0, 100 mM NaCl, 1 mM DTT, 0.1% (v/v) Triton X-100, O.N. \| \| 20 mM KPi pH 6.7, 8 M urea, 100 mM NaCl \| 80% \| 1) 20 mM KPi pH 6.7, 4 M urea, 100 mM NaCl, 1h 45min \| Yes \| No \| \| 2) 20 mM KPi pH 6.7, 2 M urea, 100 mM NaCl, 1h 45min \| \| 3) 20 mM KPi pH 6.7, 100 mM NaCl, O.N. \| \| 20 mM sodium acetate pH 4.0, 8 M urea, 100 mM NaCl \| < 5% \| 1) 20 mM sodium acetate pH 4.0, 4 M urea, 100 mM NaCl, 1h 45min \| No \| No \| \| 2) 20 mM sodium acetate pH 4.0, 2 M urea, 100 mM NaCl, 1h 45min \| \| 3) 20 mM sodium acetate pH 4.0, 100 mM NaCl, O.N. \| \| 10 mM Tris-HCl pH 8.0, 6 M GdnHCl, 50 mM NaCl \| 85% \| 1) 10 mM Tris-HCl pH 8.0, 3 M GdnHCl, 50 mM NaCl, 1h 45min \| Yes \| No \| \| 2) 10 mM Tris-HCl pH 8.0, 1.5 M GdnHCl, 50 mM NaCl, 1h 45min \| \| 3) 10 mM Tris-HCl pH 8.0, 50 mM NaCl, O.N. \| \| 20 mM sodium acetate pH 4.0, 6 M GdnHCl, 100 mM NaCl \| 95% \| 1) 20 mM sodium acetate pH 4.0, 3 M GdnHCl, 100 mM NaCl, 1h 45min \| Yes \| No \| \| 2) 20 mM sodium acetate pH 4.0, 1.5 M GdnHCl, 100 mM NaCl, 1h 45min \| \| 3) 20 mM sodium acetate pH 4.0, 100 mM NaCl, O.N. \| \| 10 mM Tris-HCl pH 8.0, 6 M GdnTC, 50 mM NaCl \| 65% \| 1) 10 mM Tris-HCl pH 8.0, 3 M GdnTC, 50 mM NaCl, 1h 45min \| Yes \| No \| \| 2) 10 mM Tris-HCl pH 8.0, 1.5 M GdnTC, 50 mM NaCl, 1h 45min \| \| 3) 10 mM Tris-HCl pH 8.0, 50 mM NaCl, O.N. \| \| **Mild solubilization methods** \| \| \| \| \| \| \| Sonication \| 50 mM Tris-HCl pH 7.5, 300 mM NaCl, 3% (v/v) Triton X-100, 30 mM CHAPS, 2% (w/v) NLS \| < 5% \| / \| / \| Traces \| \| 50 mM Tris-HCl pH 7.5, 300 mM NaCl, 4% (v/v) Triton X-100, 40 mM CHAPS, 2% (w/v) NLS \| 5-10% \| / \| / \| Traces \| \| 50 mM Tris-HCl pH 7.5, 300 mM NaCl, 8% (v/v) Triton X-100, 80 mM CHAPS, 4% (w/v) NLS \| 5-10% \| / \| / \| Traces \| \| 20 mM KPi pH 6.5, 300 mM NaCl, 15% (v/v) Triton X-100, 150 mM CHAPS, 10% (w/v) NLS \| < 5% \| / \| / \| No \| \| 50 mM Tris-HCl pH 8.5, 10% (w/v) sucrose, 0.5 mM EDTA, 1 M urea, 6 M *n*-propanol \| < 5% \| 100 mM sodium acetate pH 5.0, O.N. \| No \| No \| \| Osmotic shock and sonication \| 10 mM HCl \| > 90% \| No \| Yes \| \| 100 mM HCl \| 20% \| No \| Yes \| \| 50 mM formic acid \| 40% \| Yes \| No \| \| 10 mM lactic acid \| 80% \| No \| Yes \| \| 100 mM lactic acid \| 60% \| No \| Yes \| |
| --- | --- | --- | --- | --- | --- | --- | --- | --- | --- | --- | --- | --- | --- | --- | --- | --- | --- | --- | --- | --- | --- | --- | --- | --- | --- | --- | --- | --- | --- | --- | --- | --- | --- | --- | --- | --- | --- | --- | --- | --- | --- | --- | --- | --- | --- | --- | --- | --- | --- | --- | --- | --- | --- | --- | --- | --- | --- | --- | --- | --- | --- | --- | --- | --- | --- | --- | --- | --- | --- | --- | --- | --- | --- | --- | --- | --- | --- | --- | --- | --- | --- | --- | --- | --- | --- | --- | --- | --- | --- | --- | --- | --- | --- | --- | --- | --- | --- | --- | --- | --- | --- | --- | --- | --- | --- | --- | --- | --- | --- | --- | --- | --- | --- | --- | --- | --- | --- | --- | --- | --- | --- | --- | --- | --- | --- | --- | --- | --- | --- | --- | --- | --- | --- | --- | --- | --- | --- | --- | --- | --- | --- | --- | --- |

Protein solubilization/precipitation was estimated by SDS-PAGE analysis and enzyme activity was measured by the fluorimetric assay on 4-MU-(GlcNAc)_2_.

Abbreviations: KPi = phosphate buffer; EDTA = ethylenediaminetetraacetic acid; CHAPS = 3-[(3-cholamidopropyl)dimethylammonium]-1-propanesulfonate; NLS = *N*-lauroylsarcosine; DTT = dithiothreitol; GdnHCl = guanidium hydrochloride; GdnTC = guanidium thiocyanate; O.N. = over-night.

| **Table S2** **Affinity chromatography (AC), ion exchange chromatography (IEC) and hydrophobic interaction chromatography (HIC) pilot experiments for Chi18H8 purification** | | | | | | |
| --- | --- | --- | --- | --- | --- | --- |
| **Resin type** | **Chi18H8 buffer** | **Activation buffer** | **Equilibration buffer** | **Chi18H8 activity in the flow through (%)** | **Elution buffer** | **Chi18H8 activity in the eluate (%)** |
| HiTrap^TM^ Chelating HP (GE Healthcare) | 100 mM HEPES pH 5.6 | 100 mM HEPES pH 5.6, 20 mM imidazole, 500 mM NaCl | 100 mM HEPES pH 5.6, 20 mM imidazole, 500 mM NaCl | 100 | 100 mM HEPES pH 5.6, 250 mM imidazole, 500 mM NaCl | No |
|  | 50 mM sodium acetate pH 5.0 | 50 mM sodium acetate pH 5.0, 20 mM imidazole, 100 mM NaCl | 50 mM sodium acetate pH 5.0, 20 mM imidazole, 100 mM NaCl | 100 | 50 mM sodium acetate pH 5.0, 500 mM imidazole, 100 mM NaCl | No |
|  |  | 50 mM sodium acetate pH 5.0, 5% (w/v) glycerol | 50 mM sodium acetate pH 5.0, 5% (w/v) glycerol | 100 | 50 mM sodium acetate pH 5.0, 5% (w/v) glycerol, 500 mM imidazole | No |
| HiTrap^TM^ SP FF (GE Healthcare) | 10 mM lactic acid | 50 mM lactic acid | 50 mM lactic acid, 5 mM NaCl | 100 | 50 mM lactic acid, 0.5 M NaCl | No |
|  | 25 mM acetic acid pH 4.0 | 25 mM acetic acid pH 4.0 | 25 mM acetic acid pH 4.0 | 100 | 25 mM acetic acid pH 4.0, 1 M NaCl | No |
| DEAE-Sephadex A-25 (Pharmacia) | 100 mM HEPES pH 5.6 | 100 mM HEPES pH 5.6 | 100 mM HEPES pH 5.6 | 100 | 100 mM HEPES pH 5.6, 1 M NaCl | No |
| DEAE-Sephadex A-50 (Pharmacia) |  |  |  | 100 |  | No |
| DEAE-Cellulose (Sigma-Aldrich) |  |  |  | 100 |  | No |
| Diaion WA11 (Resindion) |  | 100 mM NaOH |  | 100 |  | No |
| Cellex-E (Bio-Rad) |  |  |  | 100 |  | No |
| Amberlite IRA-67 (BDH) |  |  |  | 100 |  | No |
| Amberlite IRA-900 (Alfa Aesar) |  |  |  | 100 |  | No |
| Amberlite IRA-904 (Alfa Aesar) |  |  |  | 100 |  | No |
| Dowex 1X8 (Fluka) |  |  |  | 100 |  | No |
| Chelex 100 (Bio-Rad) |  | 100 mM HEPES pH 5.6 |  | 100 |  | No |
| Dowex rg 50WX2 (Alfa Aesar) | 10 mM calcium lactate pH 6.6 | 100 mM HCl | 10 mM calcium lactate pH 6.6 | 87 | 10 mM calcium lactate pH 6.6, 1 M NaCl | No |
| Chelex 100 (Bio-Rad) |  | 100 mM lactic acid |  | 85 |  | No |
| CF11 (Whatman) |  |  |  | 100 |  | No |
| Cellex CM (Bio-Rad) |  |  |  | 70 |  | No |
| Diaion WA11 (Resindion) |  |  |  | 61 |  | No |
| P11 (Whatman) |  |  |  | 0 |  | No |
| Diaion WA11 (Resindion) § | 100 mM HEPES pH 5.6 | Water / methanol 1:1 | 100 mM HEPES pH 5.6 | 100 | 100 mM HEPES pH 5.6, 50% (v/v) ethanol | No |
|  |  |  |  | 100 | 50% (v/v) ethanol | No |
| HP20SS (Resindion) |  |  |  | 0 | 100 mM HEPES pH 5.6, 50% (v/v) ethanol | No |
|  |  |  |  | 0 | 50-80% (v/v) ethanol | No |

§ The resin was used in HIC mode due to its styrenic matrix

Chi18H8 activity was measured by the fluorimetric assay on 4-MU-(GlcNAc)_2_ in the flow-through and in the eluate fractions.

Abbreviations: HEPES = 4-(2-hydroxyethyl)-1-piperazineethanesulfonic acid; HP = high performance; FF = fast-flow; SP = Sepharose; DEAE = diethylaminoethanol.
