# Supplementary material for: Production and characterization of a novel antifungal chitinase identified by functional screening of a suppressive-soil metagenome
Source: Microb Cell Fact. 2017 Jan 31;16:16. doi: 10.1186/s12934-017-0634-8 (PMC5282697; doi:10.1186/s12934-017-0634-8)
Supplement: Supplementary file 2 — Additional file 2. Screening of methods for recovery and solubilization of Chi18H8 from Escherichia coli inclusion bodies (IBs) and its purification. Table S1. Different protocols used for isolation and solubilization of IBs and, eventually, for refolding Chi18H8. Table S2. Affinity chromatography (AC), ion exchange chromatography (IEC) and hydrophobic interaction chromatography (HIC) pilot experiments for Chi18H8 purification. [file 12934_2017_634_MOESM2_ESM.docx]

**Supplementary materials. Additional File 2.**

**Figure S1 Evaluation of Chi18H8 antifungal activity in liquid assays.**

Growth in liquid assay of (**a**) *Fusarium graminearum* ATCC 46779*,* and (**b**) *Rhizoctonia solani* ATCC 10183*,* respectively, in the presence of increasing concentrations of Chi18H8, in triplicate. Values show dry weight (mg) of fungal plugs normalized against dry weight of agar plug without fungi. Samples with boiled Chi18H8 were used as negative controls.

**
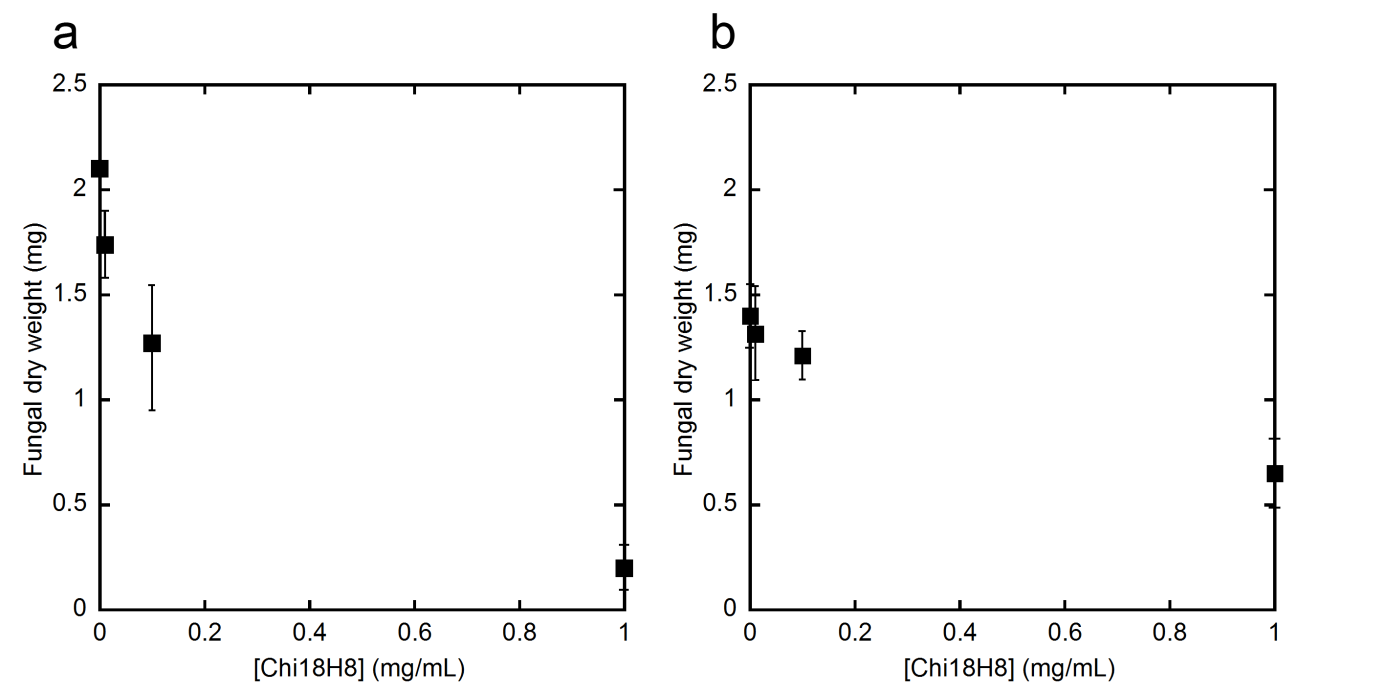
**
